# Humanized Mice as Unique Tools for Human-Specific Studies

**DOI:** 10.1007/s00005-018-0506-x

**Published:** 2018-02-07

**Authors:** Kylie Su Mei Yong, Zhisheng Her, Qingfeng Chen

**Affiliations:** 1 Institute of Molecular and Cell Biology, Agency for Science, Technology and Research (A*STAR), Proteos, 61 Biopolis Drive, Singapore, 138673 Singapore; 2 Department of Biochemistry, Yong Loo Lin School of Medicine, National University of Singapore, Singapore, 117596 Singapore; 3 Department of Microbiology and Immunology, Yong Loo Lin School of Medicine, National University of Singapore, Singapore, 117545 Singapore; 4 Key Laboratory for Major Obstetric Diseases of Guangdong Province, The Third Affiliated Hospital of Guangzhou Medical University, Guangzhou, 510150 China

**Keywords:** Humanized mice, Human specificity, Precision therapy, Human diseases, Drug testing

## Abstract

With an increasing human population, medical research is pushed to progress into an era of precision therapy. Humanized mice are at the very heart of this new forefront where it is acutely required to decipher human-specific disease pathogenesis and test an array of novel therapeutics. In this review, “humanized” mice are defined as immunodeficient mouse engrafted with functional human biological systems. Over the past decade, researchers have been conscientiously making improvements on the development of humanized mice as a model to closely recapitulate disease pathogenesis and drug mechanisms in humans. Currently, literature is rife with descriptions of novel and innovative humanized mouse models that hold a significant promise to become a panacea for drug innovations to treat and control conditions such as infectious disease and cancer. This review will focus on the background of humanized mice, diseases, and human-specific therapeutics tested on this platform as well as solutions to improve humanized mice for future clinical use.

## Introduction

Fundamental understandings of many biological processes that occur in humans have evolved from experimental studies on animal models, particularly non-human rodents and non-human primates (NHPs) (Hatziioannou and Evans 2012; Phillips et al. 2014). A major technical barrier in translating these discoveries to treatments is caused by differences in the biological systems between animals and humans (Greek and Rice 2012; Mestas and Hughes 2004; Shanks et al. 2009; Van der Worp et al. 2010). For example, functional Toll-like receptor 10 (TLR10) is absent in mice (Oosting et al. 2014) and cell expression marker CD28 is expressed on 100% of CD4^+^ and CD8^+^ T cells in mice but only on 80% of CD4^+^ and 50% CD8^+^ T cells in humans (Beyersdorf et al. 2015). Due to these differences, it is common that animal models are refractory to many infectious (Bäumler and Fang 2013; Carlton et al. 2008; Fauci 1988; Pain et al. 2008; Ploss et al. 2009), therapeutic (McKenzie et al. 1995; Rehman et al. 2011), or immunomodulatory agents (Attarwala 2010; Tsoneva et al. 2017) that are human-specific.

To address the limitations of translating discoveries on non-human animal models to clinical applications, a platform known as “humanized mice” was engineered to simulate humans at a cellular and molecular level (Bosma et al. 1983; Pearson et al. 2008). Humanized mice generated in recent years encompass functional human immune systems with expansive capabilities (Rongvaux et al. 2014) and are unprecedented platforms used for understanding disease pathogenesis and evaluation of compounds to treat a variety of human diseases which include but are not limited to, cancer (Her et al. 2017; Ito et al. 2009; Miyakawa et al. 2004; Pan et al. 2017), infectious disease (Amaladoss et al. 2015; Frias-Staheli et al. 2014; Keng et al. 2015; Yajima et al. 2008), autoimmune disease (Gunawan et al. 2017; Viehmann Milam et al. 2014; Young et al. 2015; Zayoud et al. 2013), and graft-versus-host disease (GvHD) (King et al. 2008; Kirkiles-Smith et al. 2009; Tobin et al. 2013; Zhao et al. 2015).

This review covers the background of humanized mice, diseases modelled on these platforms, human-specific therapeutics tested, and suggestions for overcoming remaining challenges to improve humanized mouse models for clinical applications.

## Evolving History of Humanized Mice

There has been a constant pursuit to engineer novel immunodeficient mouse models via gene deletion or backcrossing strains with mutations in essential molecular compartments such as, T cells, B cells, macrophages, natural killer (NK) cells, cytokines, TLRs, and transcription factors (Pearson et al. 2008). The aim of introducing these mutations is to reduce murine cells and increase the engraftment of human cells and tissues to better recapitulate human immune responses (Aryee et al. 2014; Billerbeck et al. 2011; Chen et al. 2009; Rongvaux et al. 2014; Yao et al. 2016).

Tracing the roots of humanized mice, the discovery of non-human animal models xenotransplanted with cells and tissues of human origin was credited to the invention of C.B-17-*Prkdc*^*scid*^ (CB17-*scid*) mice (Bosma et al. 1983). Derived from backcrossing C57BL/Ka and BALB/c, this mouse features loss of function mutation in a gene known as protein kinase, DNA-activated, catalytic polypeptide (*PRKDC*). In normal physiological conditions, *PRKDC* is essential for resolving breaks in DNA strands during variable, diversity, and joining [V(D)J] recombination for the development of T and B cells (Blunt et al. 1996; Finnie et al. 1996; Lieber et al. 1988; Taccioli et al. 1998). Non-functional *PRKDC* gene leads to impaired development of T and B cells resulting in syndrome known as severe combined immunodeficiency (*scid*) (Bosma and Carroll 1991). Despite efforts in creating CB17-*scid* mice, this model was not used in many experiments due to the poor engraftment of human hematopoietic stem cells (HSCs) (Bosma et al. 1983).

Further research saw the transfer of *scid* mutation onto a mouse of non-obese diabetic (NOD) background, creating NOD-*scid* mice which lacked T cells, B cells, and NK cells. This mouse allowed a slightly higher level of human cell reconstitution (Van der Loo et al. 1998). However, the biggest breakthrough in humanized mice only occurred when mutant interleukin 2 receptor α (*IL2rα*) gene was introduced into NOD-*scid* mice, creating NOD-*scid*-γc^null^ mice (NSG or NOG), which exhibited defective mouse cytokines IL-2, IL-4, IL-7, IL-9, and IL-15 (Ishikawa et al. 2005; Ito et al. 2002; Shultz et al. 2005). Knock-out of recombination activating gene (*RAG*) 1 or 2 (*RAG1*^null^ and *RAG2*^null^) caused even greater immunodeficiencies including an absence of NK cells, T cells, B cells, and impaired macrophage and dendritic cell (DC) subsets (Harris and Badowski 2014; Watanabe et al. 2007). However, an absence of human leukocyte antigen (HLA) in these models resulted in engrafted human pre-T cells being “educated” and selected on mouse thymic epithelium and major histocompatibility complexes (MHCs) (Shultz et al. 2010). Due to this limitation, engrafted human T cells were unable to recognise human antigen-presenting cells, and hence, these mice had impaired immunoglobulin (Ig) class switching and disorganised secondary lymphoid structures (Shultz et al. 2010, 2012). To overcome this hurdle, HLA class I and II transgenes were added into NSG mice allowing the development of human T-cell repertoires and responses (Brehm et al. 2013; Shultz et al. 2010).

Improved models of immunodeficient mice enabled an increase in well-differentiated multilineage human hematopoietic cells, high levels of functional human cell reconstitution and an ability to be engrafted with tissues such as thymus, skin, liver, islets, solid tumors, and blood cancers (Ito et al. 2002). These inventions cascaded into a series of immunodeficient mice and their variants (BRG, NOG, NRG) (Ali et al. 2012; Grover et al. 2017; Ishikawa et al. 2005; Katano et al. 2014; Koboziev et al. 2015; Shultz et al. 2005) being innovated which enabled in-depth analysis in research areas, such as human hematopoiesis (Rongvaux et al. 2011; Yong et al. 2016), innate and adaptive immunity (Brehm et al. 2010; Pearson et al. 2008), autoimmunity (Gunawan et al. 2017; Viehmann Milam et al. 2014), infectious disease (Keng et al. 2015; Lüdtke et al. 2015; Wege et al. 2012), cancer biology (Chang et al. 2015; Her et al. 2017; Morton et al. 2016), and GvHD (King et al. 2008; Kirkiles-Smith et al. 2009; Zhao et al. 2015), in-turn, facilitating the development of therapeutic agents and novel vaccines. An overview of genotypic and physiological characteristics of each model is outlined in Tables 1 and 2.

**Table 1:** Platforms for human immune system engrafted mice

Name	C.B-17-*scid*	NOD-*scid*	BRG	NOG	NSG™, NOD-*scid-*γ	NRG, NOD *Rag*-γ
Nomenclature	C.B-*Igh-1*^*b*^/IcrTac-*Prkdc*^*scid*^	NOD.CB17-*Prkdc*^*scid*^/J	C.Cg-*Rag2*^*tm1Fwa*^ *Il2rg*^*tm1Sug*^/JicTac	NOD.Cg-*Prkdc*^*scid*^ *Il2rg*^*tm1Sug*^/JicTac	NOD.*Cg-Prkdc*^*scid*^ *Il2rg*^*tm1Wjl*^/SzJ	NOD.*Cg-Rag1*^*tm1Mom*^ *Il2rg*^*tm1Wjl*^/SzJ
Engraftment method for humanization	HSPCs BM cells Spleen cells	HSPCs PBMCs Thymus and liver under kidney capsule with matching engraftment of HSPCs from FL Cancer derived from patients and cell lines	HSPCs PBMCs	HSPCs PBMCs Thymus and liver under kidney capsule with matching engraftment of HSPCs from FL Cancer derived from patient and cell lines	HSPCs PBMCs Thymus and liver under kidney capsule with matching engraftment of HSPCs from FL Cancer derived from patients and cell lines	HSPCs PBMCs Thymus and liver under kidney capsule with matching engraftment of HSPCs from FL Cancer derived from patients and cell lines
Limitations	Low tolerance for irradiation Intact innate immune system Rejection of engraftments Spontaneous development of thymic lymphomas Short lifespan	Low tolerance for irradiation Spontaneous development of thymic lymphomas Not all cancers can be engrafted	Spontaneous development of thymic lymphomas	Low tolerance for irradiation Not all cancers can be engrafted High occurrence of tumor metastasis	Low tolerance for irradiation Spontaneous development of thymic lymphomas Not all cancers can be engrafted	Requires a higher dose of irradiation Not all cancers can be engrafted
Applications	GvHD	Autoimmune type I diabetes Oncological studies	Immune system Infectious diseases Oncological studies	Stem cells Immune system Infectious diseases Oncological studies Drug tests	Stem cells Immune system Infectious diseases Oncological studies Drug tests	Stem cells Immune system Infectious diseases Oncological studies Drug tests
Dendritic cells	Yes	Impaired	Impaired	Impaired	Impaired	Impaired
Macrophages	Yes	Impaired	Impaired	Impaired	Impaired	Impaired
NK cells	Yes	No	No	No	No	No
Mature B cells	No	No	No	No	No	No
Mature T cells	No	No	No	No	No	No
Complement	Yes	No	No	No	No	No
Leakiness	Low	Low	No	No	Low	No
Irradiation tolerance	Low	Low	High	Low	Low	High
Lymphoma incidence	High	High	Low	No	No	Low
Median lifespan	< 12 months	< 10 months	Not determined	> 18 months	> 18 months	Not determined
References	Schneider et al. (1997) Sheng-Tanner et al. (2000) Xia et al. (2006)	Bastide et al. (2002) Brehm et al. (2013)	Traggiai et al. (2004) Ali et al. (2012) Akkina (2013)	Watanabe et al. (2009) Akkina (2013)	Yong et al. (2016) Her et al. (2017)	Harris et al. (2013) Shultz et al. (2012) Maykel et al. (2014)

**Table 2:** Platforms for human immune system engrafted mice

Name	HuNOG-EXL	NSG-SGM3	NSG-HLA-A2	NSG-Ab DR4	MISTRG	NSGW41
Nomenclature	NOD.*Cg*-*Prkdc*^scid^ *Il2rg*^*tm1*^Sug Tg(SV40/HTLV-IL3, CSF2)10-7Jic/JicTac	NOD.*Cg*-*Prkdc*^*scid*^ *Il2rg*^*tm1Wjl*^ Tg(CMV, IL3, CSF2, KITLG)1Eav/MloySzJ	NOD.Cg-*Prkdc*^*scid*^ *Il2rg*^*tm1Wjl*^ Tg(HLA-A2.1)1Enge/SzJ	NOD.Cg-*Prkdc*^*scid*^ *Il2rg*^*tm1Wjl*^ *H2-Ab1*^*tm1Gru*^ Tg(HLA-DRB1)31Dmz/SzJ	C;129S4-*Rag2*^*tm1.1Flv*^*Csf1*^*tm1(CSF1)Flv*^*Csf2*/*Il3*^*tm1.1(CSF2,IL3)Flv*^*Thpo*^*tm1.1(TPO)Flv*^*Il2rg*^*tm1.1Flv*^Tg(SIRPA)1Flv/J	NOD.Cg-*Kit*^*W*−*41J*^ *Prkdc*^*scid*^ *Il2rg*^*tm1Wjl*^/WaskJ
Engraftment method for humanization	HSPCs PBMCs Thymus and liver under kidney capsule with matching engraftment of HSPCs from FL Cancer derived from patients and cell lines	HSPCs PBMCs Thymus and liver under kidney capsule with matching engraftment of HSPCs from FL Cancer derived from patients and cell lines	HSPCs PBMCs	PBMCs	HSPCs Human melanoma cell line (Me290)	HSPCs
Limitations	Not all cancers can be engrafted Mice with high chimeric ratio develop anemia after engraftment	Human cell engraftment does not last more than five months	Low tolerance for irradiation	Low CD45^+^ human cell engraftment compared to NSG mice	Short lifespan post-engraftment (~ 10–12 weeks) but may be prolonged by avoiding irradiation, using less potent and lower number of stem cells	Not reported
Applications	Stem cells Immune system Infectious diseases Oncological studies Drug tests	Stem cells Immune system Infectious diseases Oncological studies Drug tests	Immune system Oncological studies Vaccine development	GvHD	Stem cells Immune system Oncological studies	Stem cells
Dendritic cells	Impaired	Impaired	Impaired	Impaired	Impaired	Impaired
Macrophages	Impaired	Impaired	Impaired	Impaired	Impaired	Impaired
NK cells	No	No	No	No	No	No
Mature B cells	No	No	No	No	No	No
Mature T cells	No	No	No	No	No	No
Complement	No	No	No	No	No	No
Leakiness	No	No	Low	Low	No	No
Irradiation tolerance	Not determined	Not determined	Low	High	Low	Low
Lymphoma incidence	Not determined	Not determined	No	No	Not determined	Not determined
Median lifespan	> 7 months	> 4 months	> 18 months	Not determined	Not determined	Not determined
References	Fukuchi et al. (1998) Ito et al. (2013)	Billerbeck et al. (2011)	Whitfield-Larry et al. (2011) Patton et al. (2015)	Covassin et al. (2011)	Rongvaux et al. (2014)	Rahmig et al. (2016)

*HSPCs* hematopoietic stem and progenitor cells, *FL* fetal liver, *GvHD* graft-versus-host disease, *PBMCs* peripheral blood mononuclear cells, *BM* bone marrow

The conventional ways to engraft immunodeficient mice with functional human cells include, intravenous (i.v.) injection of human peripheral blood mononuclear cells (PBMCs) into mice (Hu-PBL-*scid*) (Duchosal et al. 1992; Harui et al. 2011; King et al. 2008; Tary-Lehmann et al. 1995), injecting CD34^+^ HSCs obtained from human fetal liver (FL), umbilical cord blood (UBC), bone marrow (BM) or granulocyte-colony-stimulating factor (G-CSF) mobilised peripheral blood (Hu-SRC-*scid*) (Brehm et al. 2010; Chen et al. 2009, 2012, 2015; Keng et al. 2015; Yong et al. 2016), or i.v. injection of FL HSCs and BM cells paired with transplantation of matching FL and thymus under the kidney capsule to obtain a BM/liver/thymus (BLT) mouse model (Brainard et al. 2009; Covassin et al. 2013; Denton et al. 2008; Lan et al. 2004, 2006; Melkus et al. 2006; Tonomura et al. 2008). Advantages and drawbacks of each method are compared in Table 3. However, despite efforts in optimising humanized mice, critical challenges that remain include: limited fetal samples due to ethical restrictions (Geraghty et al. 2014; Kapp 2006), absence of erythrocytes and neutrophils within reconstituted human immune system (Hu et al. 2011), low and impaired human myeloid cells, dominance of immature B cells (Chen et al. 2012; Lang et al. 2013), and minimal production of antigen-specific IgG class antibodies in humanized mice (Jangalwe et al. 2016).

**Table 3:** Methods used to establish humanized mouse models

Model	Human PBMCs engrafted into immunodeficient mice	Human HSCs engrafted into immunodeficient mice	Human HSCs, BM, liver, and thymus engrafted into immunodeficient mice
Alternative name	Hu-PBL-*scid*	Hu-SRC-*scid*	BLT
Source of cells	Obtained from consented adult donors	FL UBC BM G-CSF mobilised peripheral blood	FL Fetal BM Fetal thymus
Method of engraftment	Intravenous injection of mice	Intrahepatic injection of newborn mice within 72 h of birth Intravenous injection of mice	Implantation of liver and thymus under the kidney capsule Transplantation of matching HSCs obtained from FL
Advantages	Easy techniques applied Fast to establish Presence of functional immune cells such as memory T cells Excellent in modelling GvHD	Multilineage development of hematopoietic cells Generation of a naïve immune system Injection to pups increase human cell reconstitution	Complete and fully functional human immune system HLA-restricted T cells Development of a mucosal system similar to humans Highest level of human cell reconstitution among all the models
Drawbacks	Lack B and myeloid cell engraftment Engrafted T cells are activated May develop GvHD Only suitable for short-term experiments (< 3 months)	Cell differentiation takes a minimum of 10 weeks Engrafted human T cells are H2 restricted Contains low levels of human RBCs, polymorphonuclear leukocytes, and megakaryocytes	Time-consuming and difficult as surgical implantation is required Cell differentiation takes a minimum of 10 weeks Weak immune responses to xenobiotics Poor class switching May develop GvHD

*BLT* bone marrow/liver/thymus, *HSCs* hematopoietic stem cells, *FL* fetal liver, *GvHD* graft-versus-host disease, *PBMCs* peripheral blood mononuclear cells, *UBC* umbilical cord blood, *BM* bone marrow, *G-CSF* granulocyte-colony-stimulating factor, *RBC* red blood cells

To overcome technical barriers, a few methods to improve the functional human biological systems in mice is to inject humanized mice with recombinant proteins (Huntington et al. 2009; Van Lent et al. 2009), hydrodynamically inject DNA plasmids (Chen et al. 2009), induce lentivirus expression of cytokines (Van Lent et al. 2009), or introduce knock-in gene replacement as so to increase the repertoire of cytokines to support human cells (Billerbeck et al. 2011; Lim et al. 2017; Nicolini et al. 2004; Rongvaux et al. 2011). An example of a technique that is effective does not require complex procedures and can be readily applied in any laboratory is the injection of plasmid DNA (IL-15 and Fms-like tyrosine kinase 3/fetal liver kinase-2 (FLT3/FLK2) ligand) via hydrodynamic tail-vein injection (Chen et al. 2009). Upon application of this method, the expression levels of human cytokines were present for 2–3 weeks, while the levels of functional NK cells remained high for more than a month (Chen et al. 2009). Unlike mice induced to constitutively express cytokines which may activate cells and skew them toward unideal lineages, hydrodynamic injection enables researchers to control the exact timing of cytokine induction, allowing flexible manipulation of the model. On top of this, cytokine-stimulated NK cells expressed activation and inhibitory receptors; attacked in vitro target cells, and responded well to viral infections within an in vivo setting (Chen et al. 2009).

Another method which requires more time and resources to create but eliminates the need for cytokine plasmid injection is the use of transgenic mice with knock-in genes, encoding for cytokines. Four examples of these enhanced immunodeficient mice are, first, NOD.Cg-Prkdc^scid^ Il2rg^tm1Sug^Tg (SV40/HTLV-IL3, CSF2) 10-7Jic/JicTac (huNOG-EXL mouse), this strain of super immunodeficient mouse has a high rate of human cell engraftment and expresses both granulocyte/macrophage colony-stimulating factor (GM-CSF) and human IL-3 cytokines, controlled by SV40 promoter, which induces myeloid reconstitution and differentiation.

Second, NOD.Cg-*Prkdc*^*scid*^ *Il2rg*^*tm1Wjl*^ Tg (CMV-IL3, CSF2, KITLG) 1Eav/MloySzJ (NSG-SGM3 mouse) are knock-in mice expressing IL-3, GM-CSF and stem cell factor (SCF) under the control of human-specific cytomegalovirus (CMV) (Billerbeck et al. 2011; Yao et al. 2016). Even though this combination of genes supports human HSC engraftment, formation of myeloid leukocytes, and reduces B-lymphopoiesis post-BM transplantation this model lacks an improved red blood cell (RBC) reconstitution and the presence of SCF may destructively affect human stem cell compartments by supporting the growth and competitive repopulation of mouse cells (Billerbeck et al. 2011; Yao et al. 2016).

Third, C;129S4-*Rag2*^*tm1.1Flv*^ *Csf1*^*tm1(CSF1)Flv*^ *Csf2*/*Il3*^*tm1.1(CSF2,IL3)Flv*^ *Thpo*^*tm1.1(TPO)Flv*^ *Il2rg*^*tm1.1Flv*^Tg (*SIRP*α) 1Flv/J (MISTRG mouse) was designed to support a greater level of human cell reconstitution, particularly in the myeloid compartment by transgenically inducing human GM-CSF, IL-3, macrophage colony-stimulating factor (M-CSF), thrombopoietin (TPO), and signal-regulatory protein alpha (*SIRP*α). *SIRP*α produces anti-phagocytic signals upon interaction with human CD47 cells which inhibits murine macrophages from phagocytosing human cells (Rongvaux et al. 2014). However, due to poor erythropoiesis of both mouse and human cells especially post-irradiation preconditioning, MISTRG mice developed severe anemia resulting in its short lifespan and was eventually discontinued commercially (Rongvaux et al. 2014).

Fourth, NOD.Cg-*Kit*^*W*−*41J*^ *Prkdc*^*scid*^ *Il2rg*^*tm1Wjl*^/WaskJ (NSGW41) was created to overcome a lack of erythro-megakaryopoiesis in humanized mouse models. Without the need for irradiation, this KIT-deficient mouse demonstrated improved erythropoiesis and platelet formation as compared to other models of mice (Cosgun et al. 2014; Rahmig et al. 2016). After reconstitution, significant numbers of mature thrombocytes were present in the peripheral blood while human erythroblasts were seen in the BM. In addition, the morphology, composition, and enucleation ability of *de novo* generated human erythroblasts were similar with those in the human BM (Rahmig et al. 2016). However, as this model is relatively new, more studies are needed to further characterise the advances and limitations of this platform. Details of immunodeficient mouse models are listed in Tables 1 and 2. As existing models are far from perfect, it is important to work on components that enhance cell–cell interactions, support differentiation, and induce maturation of human cells, particularly that of myeloid and B cell compartments to create a model that faithfully recapitulates the human immune system.

## Models of Human Diseases Established on Humanized Mice

The introduction of humanized mice provides immeasurable opportunities to advance medical research. These increasingly important pre-clinical models are not only easy to handle due to their small sizes, but they also have short reproductive cycles, an exceptional ability to produce a large number of young and are relatively affordable to maintain in animal facilities as they do not require highly specialised infrastructures that are used by NHPs (Fischer and Austad 2011). In addition, humanized mice allow human-specific pathogens to infect and replicate within them and are able to develop functional human-specific immune responses to an array of diseases.

Many mechanisms underlying diseases are not completely dissected; therefore, utilization of humanized mice allows researchers to understand important factors that facilitate the development of medical issues including infectious disease, cancer, autoimmunity, and GvHD. Currently, a mouse model that completely mimics every single human disease does not exist; therefore, research aims such as the consideration of specific parameters to be analyzed including genotype, phenotype of the model, and scientific budget must be thought through carefully to select a suitable platform.

### Infectious Disease

Since the invention of humanized mice, multitudinous attempts have been made to recapitulate infectious diseases within these mice. A particular human-specific infectious pathogen that has been successfully studied on humanized mice is a retrovirus known as human immunodeficiency virus (HIV) (Araínga et al. 2016; Berges and Rowan 2011; Choudhary et al. 2009; Duyne et al. 2011; Li et al. 2014). Before humanized mice were introduced, the only non-human animal model available for dissecting HIV pathogenesis was the chimpanzee (Vanden Haesevelde et al. 1996). Because of cellular and molecular differences between HIV pathogenesis in humans and chimpanzees, restricted tropism of HIV and high-expense of using NHPs, the small, cost-effective, and widely available humanized mice were used in place of the NHPs (Denton and Garcia 2011; Hatziioannou and Evans 2012; Miller et al. 2000).

Humanized mice infected with HIV recapitulated the disease’s progression, latency and virology, permitted long-term immunological studies and helped identify crucial factors such as viral infectivity factor, viral protein u, and negative factor which are essential for in vivo HIV replication (Yamada et al. 2015).

Of all the models (Hu-PBL-*scid*, Hu-SRC-*scid* and BLT) (Choudhary et al. 2012; Dash et al. 2011; Gorantla et al. 2010; Ince et al. 2010; Long and Stoddart 2012; Sato et al. 2010; Zhang et al. 2011) used to characterise HIV, BLT mice (Carter et al. 2011; Denton et al. 2012; Marsden et al. 2012) had the most accurate representation of the human mucosal system (Brainard et al. 2009; Denton et al. 2010; Sun et al. 2007), allowing the study of vaginal and rectal transmission and prevention of HIV by enabling evaluations of many prophylactic therapeutics (Balazs et al. 2011), anti-HIV antibodies (Choudhary et al. 2009; Joseph et al. 2010), and cellular therapeutic inventions for inhibiting or eliminating HIV (Holt et al. 2010; Kumar et al. 2008; Neff et al. 2011; Shimizu et al. 2010).

Humanized mouse model with a fully functional human immune system has also been infected with Dengue virus (DENV) (Frias-Staheli et al. 2014; Kuruvilla et al. 2007; Sridharan et al. 2013; Subramanya et al. 2010). These mice demonstrated fever, rash, viremia, erythema, thrombocytopenia, and production of anti-DENV IgM, IgG and a range of cytokines as observed in patients (Mota and Rico-Hesse 2009, 2011). Another human-specific infectious pathogen studied on humanized mice, Plasmodium falciparum, is a causative agent of malaria (Amaladoss et al. 2015; Carlton et al. 2008; Chen et al. 2014; Good et al. 2015; Jiménez-Díaz et al. 2009; Soulard et al. 2015; Vaughan et al. 2012). For years, our understanding of malaria had been impeded by the lack of human-specific small animal models which can be infected by highly host-specific human Plasmodium species (Amaladoss et al. 2015; Chen et al. 2014; Pain et al. 2008) to recapitulate both erythrocytic and immunological disease pathogenesis observed in patients. Due to this, most in vivo experimental studies of malaria were conducted in rodents with mouse or rat-specific Plasmodium strains (Goodman et al. 2013). Differences in invasion and disease pathology between human and rodent parasite species hindered the translation of findings and evaluation of new therapeutics from rodents to humans (Amaladoss et al. 2015; Chen et al. 2014). This challenge has been tackled by incorporating RBC supplemented, immune cell-optimised (enhanced by hydrodynamic expression of human cytokines, IL-15, and FLT3/FLK2 ligand) humanized mice that supports multiple cycles of P. falciparum infection (Amaladoss et al. 2015; Chen et al. 2014).

Utilizing this model, research teams were able to identify the importance of human NK cells, DCs, and B cells in the control of parasitemia. Notably, how NK cells preferentially interacts with infected RBCs (iRBCs), resulting in the activation of NK cells, release of interferon (IFN)-γ, perforin, and granzyme to lyse and eliminate iRBCs in a contact-dependent manner and the importance of adhesion molecule lymphocyte-associated antigen-1 and DNAX accessory molecule-1 which are required for NK cell interaction and clearance of iRBCs (Amaladoss et al. 2015; Chen et al. 2014). Besides facilitating the understanding of human immune responses to Malaria infection, the use of humanized mice also assists in evaluation of new therapeutics and vaccines (Good et al. 2015; Tsuji et al. 1995).

In addition to the human immune system, recent progress has been made to introduce humanization of the liver in humanized mice to support the study of hepatotropic pathogens such as hepatitis B virus and hepatitis C virus (HCV) (Bility et al. 2012; Keng et al. 2015; Strick-Marchand et al. 2015; Tan-Garcia et al. 2017; Washburn et al. 2011). It has been shown that these new humanized mice could be infected with human strains of hepatitis viruses and exhibit leukocyte infiltrations, liver inflammation, fibrosis, cirrhosis, and elevated cytokines similar to HCV-infected patients (Bility et al. 2014; Keng et al. 2015; Tan-Garcia et al. 2017; Washburn et al. 2011). Mouse models with human liver cells and matched human immune system provides an important platform for understanding disease pathogenesis of hepatitis viruses through human-specific cytokines, chemokines and immune cell regulations involved, potentially translating this knowledge into creation of anti-fibrotic and immune-modulatory therapeutics (Bae et al. 2015; Keng et al. 2015).

Other examples of infectious pathogens studied on humanized mice include, *Mycobacterium tuberculosis* (Calderon et al. 2013; Nusbaum et al. 2016), influenza (Yu et al. 2008; Zheng et al. 2015), *Borrelia hermsii* (Vuyyuru et al. 2011), human CMV (Daenthanasanmak et al. 2015; Smith et al. 2010), Ebola virus (Bird et al. 2016; Lüdtke et al. 2015), Epstein-Barr virus (Cocco et al. 2008; Sato et al. 2011; Yajima et al. 2008) and Kaposi’s sarcoma-associated herpesvirus (Boss et al. 2011; Chang et al. 2009; Wang et al. 2014). Further details on infectious pathogens that have been studied using humanized mice as a platform are detailed in Table 4.

**Table 4:** Infectious diseases modelled in humanized mice

Infectious disease	Model	Main findings	References
*Borrelia hermsii*	Newborn NSG engrafted with human CD34^+^ UBC cells within 48 h of birth and intravenously or intraperitoneally infected with *B. hermsii*	Similar to clinical scenarios, infection of humanized mice with *B. hermsii* resulted in recurrent episodes of bacteremia which was resolved with *B. hermsii* specific IgM production. Anti-*B. hermsii* responses were diminished and persistent bacteremia recurred upon administration of anti-human CD20 antibody	Vuyyuru et al. (2011)
DENV	NOD/*scid* engrafted with human fetal thymus and liver tissue under the kidney capsule and intravenously injected with CD34^+^ human FL cells to create huBLT mice. Mice were intravenously infected with DENV-2	Intravenous inoculation of DENV-2 resulted in sustained viremia and infection of leukocytes in lymphoid and non-lymphoid organs. Serum cytokine levels and DENV-2-neutralising human IgM antibodies were detected in infected mice. In re-stimulation with DENV-infected DCs, in vivo primed T cells were activated and had effector functions	Frias-Staheli et al. (2014)
Ebola virus	NSG-A2 intravenously (retro-orbital) injected with human CD34^+^ UBC from HLA-A2 donors and intraperitoneally infected with Ebola virus	Similar to clinical scenarios, mice showed signs of viremia, cell damage, liver steatosis, and hemorrhage	Lüdtke et al. (2015)
EBV	NOG mice intravenously injected with human CD34^+^ UBC and EBV	B cell lymphoproliferative disorder was observed with high dose of EBV. Low dose of EBV resulted in asymptomatic persistent infection, increased levels of CD8^+^ T in the peripheral blood, EBV-specific T cell responses and IgM specific to EBV-encoded protein BFRF3	Yajima et al. (2008)
HBV	NSG-A2 mice were intrahepatically injected with autologous CD34^+^ HSC and hepatic progenitor cells to create A2/NSG-hu HSC/Hep mice. These mice were intravenously infected with clinical isolates of HBV	Mice were able to demonstrate persistent infection for up to 4 months after HBV inoculation. Similar to clinical scenarios, chronic liver inflammation, liver fibrosis and immune responses were observed in infected mice. Neutralising antibody (anti-HBsAg scFv) was able inhibit liver disease	Bility et al. (2014)
HCV	Newborn NSG were intrahepatically injected with human CD34^+^ FL cells within 72 h of birth and intravenously infected with HCV	Humanized mice were able to support HCV infection and demonstrated clinical symptoms and immune responses (innate and adaptive) commonly observed in HCV-infected patients	Keng et al. (2015)
hAdV	HLA-A2 mice were engrafted with autologous human CD34^+^ HSPCs from UCB via intra-orbital injection and intravenously infected with hAdV	Humanized mice recapitulated the pathology of acute and persistent hAdV infection. In acute infection, high mortality, weight loss, liver pathology and expression of viral protein within organs were observed. Chronic infection was asymptomatic and resulted in the development of hAdV-specific adaptive immunity and expression of early viral genes within the BM	Rodríguez et al. (2017)
hCMV	NRG mice engrafted with CD34^+^ human cells isolated from adult PBMCs and UBC and infected with hCMV	When a tricistronic integrase-defective lentiviral vector (co-expressing GM-CSF, IFN-α, and hCMV pp65 antigen) which induced self-differentiation of monocytes in PBMCs and UCB into DCs with pp65 (“SmyleDCpp65”) was administered, humanized mice infected with hCMV demonstrated remodeling of LNs, upregulation of thymopoiesis in CD4^+^ and CD8^+^ T cell precursors, polyclonal effector memory CD8^+^ T cells expansion in blood, spleen, and BM, PP65-specific CTL, and IgG responses	Daenthanasanmak et al. (2015)
HIV	Newborn NSG intrahepatically injected with CD34^+^ human FL cells and infected with HIV-1_ADA_ via intraperitoneal injection	Cell distribution and HIV viral life cycle were dependent on tissue compartment and time of infection. HIV-1 in cells was found as forms of integrated DNA and multi- and un-spliced RNA	Araínga et al. (2016)
HTLV1	NOG mice engrafted with human CD133^+^ UBC cells by IBMI) to create IBMI-huNOG mice which were intraperitoneally infected with HTLV-1	Infected mice recapitulated symptoms of adult T-cell leukemia and HTLV-1-specific adaptive immune responses including, elevation of CD4^+^ T cells, and signs of atypical lymphocytes with lobulated nuclei	Tezuka et al. (2014)
Influenza	Rag2^−/−^γc^−/−^ mice intraperitoneally injected with human PBMCs and Intranasally infected with Influenza	Intraperitoneal injection of pamidronate induced Vδ2-T cells to secrete IFN-γ and kill virus infected host cells which helped to control viral replication and suppressed inflammation in lungs of H7N9-infected mice, reducing their morbidity and mortality	Zheng et al. (2015)
KSHV	NSG mice engrafted with human fetal thymus and liver tissue under the kidney capsule and intravenously injected with CD34^+^ human FL cells to create huBLT mice. Mice were infected with KSHV via the oral mucosa	Mice were infected with KSHV via the oral mucosa and established a robust infection by targeting human macrophages and B cells	Wang et al. (2014)
Leishmania major	Newborn NSG intrahepatically injected with human CD34^+^ UBC cells and infected with Leishmania major via subcutaneous footpad injection	At the site of injection, human macrophages were infected with *Leishmania* parasites and *Leishmania*-specific human T cell responses were detected. Miltefosine reduced parasitic load and induced side-effects as observed in clinical scenarios	Wege et al. (2012)
Malaria	Newborn NSG intracardially injected with human CD34^+^ UBC cell and intravenously infected with malaria	NSG mice were supplemented human erythropoietin and IL-3 via hydrodynamic tail-vein injection. Human RBCs generated *de novo* were infected with *P. falciparum* and it was observed that different strains of parasites varied in their infection rates	Amaladoss et al. (2015)
NiV	NSG mice engrafted with human lung tissue and intragraft injected with NiV	Human fetal lung xenografts were able to form human adult lung structures. NiV replicated to high titers and infected human lung tissues resulting in the production of cytokines and chemokines including IL-6, G-CSF, and GM-CSF which commonly causes acute lung injury	Valbuena et al. (2014)
*Mycobacterium tuberculosis*	NSG mice engrafted with human fetal thymus and liver tissue under the kidney capsule and intravenously injected with CD34^+^ FL cells to create huBLT mice. These mice were intranasally infected with *tdTomato M. tuberculosis* H37Rv	Mice infected with *M. tuberculosis* demonstrated progressive bacterial infection within the lung which disseminated to the spleen and liver. Pathological analysis of the infected lung displayed obstruction of the bronchial, granulomatous lesions, caseous necrosis and crystallised cholesterol deposits. Human T cells were detected at sites of inflammation and bacterial growth, within the lung, liver, and spleen	Calderon et al. (2013)
VZV	NOD/*scid* mice engrafted with human fetal thymus and liver tissue under the kidney capsule or subcutaneously implanted with fetal skin. MRC-5 cells infected with wild-type VZV/Oka strain was injected into the implants	Varicella-zoster viral proteins were expressed in CD4^+^ and CD8^+^ T cells which have a capacity to cause viremia. Similar to clinical scenarios, skin implants infected with VZV showed lesions of varicella	Moffat et al. (1995)

*DENV* Dengue virus, *EBV* Epstein–Barr virus, *HBV* hepatitis B virus, *HCV* hepatitis C virus, *hAdV* human adenovirus, *hCMV* human cytomegalovirus, *HIV* human immunodeficiency virus, *HTLV1* human T-lymphotropic virus 1, *KSHV* Kaposi’s sarcoma-associated herpesvirus, *NiV* Nipah virus, *VZV* Varicella-zoster virus, *BLT* bone marrow/liver/thymus, *HSC* hematopoietic stem cells, *FL* fetal liver, *PBMCs* peripheral blood mononuclear cells, *UCB* umbilical cord blood, *BM* bone marrow, *GM-CSF* macrophage granulocyte-colony-stimulating factor, *IBMI* intra-BM injection, *HSPCs* hematopoietic stem and progenitor cells, *DCs* dendritic cell, *IFN* interferon, *LNs* lymph nodes, *scFv* single-chain variable fragment, *CTL* cytotoxic T lymphocyte, *RBC* red blood cell, *tdTomato* Tandem dimer Tomato

### Cancer

Immunodeficient mice that lack innate and adaptive immune cell compartments enable successful engraftment of many human tumors including tumor cell lines and primary solid and hematological tumors. Currently, there are three ways to study tumor growth and cancer immunology in humanized mice. First, tumor cell lines can be engrafted into humanized mice reconstituted with HSCs or PBMCs (Ito et al. 2009; Tsoneva et al. 2017; Wege et al. 2014). Breast cancer was modelled in mice by concurrently transplanting CD34^+^ HSCs and tumor cells into newborn mice or engrafting both PBMCs and tumor cells into BRG mice (Wege et al. 2014). In these models, human immune cells were able to traffic and infiltrate the microenvironment, enabling human tumor-immune system interactions to be studied (Wege et al. 2014). To more closely recapitulate human immune responses to tumor cell lines, MISTRG mice engrafted with CD34^+^ human FL cells were subcutaneously transplanted with a melanoma cell line, Me290 (Rongvaux et al. 2014). Similar to clinical scenarios, it was observed that myeloid cells infiltrated the tumor, numerous cells within the tumor expressed CD14 and CD163 which are commonly associated as macrophage markers, and CD163^+^ cells were most likely M2-like macrophages as they were HLA-DR^low^ and CD206^high^. It was hypothesised that tumor growth may have been mediated by M2-like macrophages that can induce cytokine production or release enzymes to promote vascularisation and immune suppression. Therefore, these mice were treated with human-vascular endothelial growth factor (VEGF) inhibitor, Avastin^®^. Humanized mice engrafted with Me290 responded to treatment by inhibiting tumor growth, suggesting that myeloid cells may support tumor growth via VEGF activity (Rongvaux et al. 2014).

Second, immunodeficient mice can be engrafted with patient-derived xenografts (PDX) (Bankert et al. 2011; Her et al. 2017; Simpson-Abelson et al. 2008). Engraftment of patient-derived acute myeloid leukemia (AML) cells into newborn NSG resulted in high levels of human cell engraftment in the peripheral blood, spleen and BM of recipient mice (Her et al. 2017). Similar to observations in the clinics, these mice also had enlarged spleens and infiltration of AML cells into multiple organs. Even though AML remained unaltered during serial transplantation, many studies with engrafted PDXs into immunodeficient mice have demonstrated that heterogeneity of parental tumor was often only maintained in primary engraftment (Cassidy et al. 2015). Over time and tumor passage, human stromal was frequently compromised by infiltration and replacement with mouse-derived cells (Cassidy et al. 2015; Maykel et al. 2014). This model is ideal for understanding stroma–tumor interactions, which is integral for tumor growth and an important target for cancer therapy.

Third, for a comprehensive study of interactions between human immune cells and tumor in vivo, immunodeficient mice should be engrafted with PDX and human immune cells (Pan et al. 2017; Roth and Harui 2015). This humanized PDX model would not only have a complete tumor microenvironment but also an ability to display heterogeneity lost in tumors (Pan et al. 2017). However, a drawback of this model is the scarcity of autologous HSCs which affects the capacity to generate cohorts for research. To overcome this challenge, HSCs isolated from UBC, FL or G-CSF mobilised PBMCs can be expanded either by transduction with tat-MYC and tat-Bcl2 fusion proteins or cultured with a validated cocktail of growth factors to induce in vitro proliferation of HSCs (Bird et al. 2014; Yong et al. 2016). An example of this model is XactMice which are engrafted with in vitro expanded HSCs and autologous PDX samples from head and neck squamous cell carcinoma patients (Morton et al. 2016). Even though these mice had low levels of humanization in their peripheral blood, they demonstrated an increase in lymphatic vessels and the presence of CD45^+^CD151^+^ cells, suggesting that these mice were able to recapitulate immune and stromal cell compartments of the tumor microenvironment (Morton et al. 2016).

While the current immunodeficient mouse strains are able to support the engraftment of most tumor cell lines, not all primary tumors for example prostate cancer can be easily engrafted (Roth and Harui 2015). Novel humanized oncological models are being innovated to address important questions on tumor-immune system interactions, mechanisms of tumor escape, therapeutic potential of immune modulation, as well as refining therapeutic solutions such as chemotherapy, NK cell therapy, checkpoint inhibitors and cytokine therapy. Tumor cell lines, and solid and hematological cancers tested on humanized mice are listed in Table 5.

**Table 5:** Cancer modelled in humanized mice

Cancer	Model	Main findings	References
Bladder	NSG mice were injected with CD34^+^ hematopoietic progenitor cells and subcutaneously engrafted with patient-derived bladder cancer cells	Major human immune cell subsets were reconstituted in humanized mice, no xenograft-versus-host disease was observed and PDX retained morphological and genetic fidelity of parental patient cancer	Pan et al. (2017)
Breast	NSG were intrahepatically engrafted with human breast carcinoma cell line (SK-BR-3)	Mice were engrafted with functional human immune system and human breast cancer cells. MHC-mismatched tumor cells resulted in activated immune cells, but no clinical signs of rejection were observed	Wege et al. (2014)
Cervical	Human cervical carcinoma cell line (C33a) was subcutaneously engrafted into *scid* mice	Herpes simplex virus type I-based oncolytic treatment in combination with radiation therapy may be an effective treatment for cervical cancer	Blank et al. (2002)
Colorectal	*Rag2*^−/−^γc^−/−^ mice were injected with human PBMCs and subcutaneously engrafted on the flank with colorectal carcinoma cell line (HT-29)	Co-administration of Urelumab and Nivolumab slowed down tumor growth by elevating activated human T lymphocytes which produced IFN-γ and decreased levels of human regulatory T cells in tumor xenografts	Sanmamed et al. (2015)
Gastric	Patient-derived xenografts of gastric cancer were subcutaneously engrafted into the right hind flank of *scid* and nude mice	Mice engrafted with patient-derived gastric cancers demonstrated identical histological and genetic diversities which corresponded to parental patient tumors	Zhang et al. (2015)
HNSCC	NSG mice were injected with expanded HSPCs and engrafted with patient-derived HNSCC	Human immune and stromal cells produced in XactMice mimics patient’s tumor microenvironment. This model was able to reverse genetic drift of tumors that usually occur after serial transplantation in non-humanized mice	Morton et al. (2016)
Kidney	NSG mice were engrafted with human RCC cell line (SKRC-59 cells) in the left subrenal capsule of their kidney	Human anti-CAIX mAbs inhibit RCC growth by halting migration and triggering immune-mediated killing of RCC. Improvements to anti-CAIX mAbs demonstrated enhanced antibody-dependent cell-mediated cytotoxicity against RCC	Chang et al. (2015)
Leukemia	Newborn NSG were intravenously engrafted with patient-derived AML cells	High levels of AML engraftment were observed in the peripheral blood, spleen and BM of recipient mice. Similar to clinical scenarios, mice had enlarged spleen and infiltration of AML cells into multiple organs. Serial transplantation did not alter AML cells	Her et al. (2017)
Lung	NSG and C.B-17-*scid* subcutaneously engrafted with patient-derived xenograft at a position caudal to the xiphoid process	NSG mice were successfully engrafted with patient-derived primary lung tumors. Mice retained parental tumor architecture such as tumor-associated leukocytes, stromal fibroblasts, and had limited xenograft-versus-host disease. Tumor-associated T cells migrated from the microenvironment of xenografts toward the lung, liver, and spleen of mice	Simpson-Abelson et al. (2008)
Lymphoma	NOG mice were subcutaneously engrafted with human PBMCs and injected with Hodgkin lymphoma cell line (L-428) or cutaneous T-cell lymphoma cell line (HH)	Anti-CCR4 mAb KM2760 demonstrated anti-tumor activity in humanized mouse models of lymphoma. Upon treatment of KM2760, tumor-infiltrating CD56^+^ NK cells were increased and T-regulatory cells were decreased	Ito et al. (2009)
Melanoma	Newborn NSG were intrahepatically injected with CD34^+^ UBC and injected with human melanoma cell lines (1935-MEL and 888-MEL)	Mice were successfully engrafted with a functional human immune system. Oncolytic vaccinia virus therapy, particularly CTLA4 scAb increased CD56^+^ NK cells and decreased virus titers	Tsoneva et al. (2017)
Myeloma	NOG mice were intravenously engrafted with human myeloma cell lines (U266)	U266 myeloma cells homed to the BM and resulted in paralysis of NOG mice	Miyakawa et al. (2004)
Ovarian	NSG mice were intraperitoneally engrafted with patient-derived xenografts of primary and metastatic ovarian solid tumor tissue and ovarian ascites fluid	Similar to clinical patients, tumors engrafted in these mice established in the omentum, ovaries, liver, spleen, uterus, and pancreas	Bankert et al. (2011)
Pancreatic	NSG mice were engrafted with patient-derived pancreatic cancer tumors by subcutaneous, intravenous or intra-pancreatic injections	Activated allogenic and autologous NK cells were able to selectively kill cancer stem cells in NSG mice engrafted with pancreatic cancer	Ames et al. (2015)
Prostate	NSG mice were injected with PBMCs with subsets of CD4^+,^ CD8^+^ and autologous DCs and subcutaneously injected with human prostate cancer cells (PC3) into the right flank	Tumor-infiltrating lymphocytes in NSG mice with a functional human immune system and prostate cancer cells were similar to clinical scenarios	Roth and Harui (2015)

*HNSCC* head and neck squamous cell carcinoma, *RCC* renal cell carcinoma, *AML* acute myeloid leukemia, *PDX* patient-derived xenografts, *mAbs* monoclonal antibodies, *scAb* single-chain antibody

### Autoimmunity

Disparities in the immune system between mice and men restrict the use of mouse models which develops spontaneous autoimmunity (Covassin et al. 2013). To overcome this challenge, Gunawan et al. (2017) engrafted PBMCs from systemic lupus erythematosus (SLE) patients to create a human-specific disease-based immune system which demonstrated that human T and B cells were present in the peripheral blood and spleen of humanized mice and were important to lupus development. Similar to patients, when these mice were treated with dexamethasone, spleen weight, and proteinuria decreased. Mice with a human immune system xenografted with patient samples allow a spectrum of disorders such as SLE (Andrade et al. 2011; Gunawan et al. 2017) and type I diabetes (Shultz et al. 2007; Unger et al. 2012; Viehmann Milam et al. 2014) to be evaluated for the identification of screening markers, retrieval of antigen-specific autoantibodies, and drug tests. Autoimmune diseases that have been studied using humanized mice as a platform are listed in Table 6.

**Table 6:** Autoimmune diseases modelled in humanized mice

Autoimmunity	Models	Main findings	References
Multiple sclerosis	NSG mice engrafted with PBMCs and injected with myelin antigens in Freund’s adjuvant and antigen-pulsed autologous DCs	Mice demonstrated subclinical CNS inflammation. Human T cells (CD4^+^ and CD8^+^) were specific to the soluble domain of myelin oligodendrocyte glycoprotein and produced proinflammatory cytokines	Zayoud et al. (2013)
SLE	NSG mice engrafted with FL HSCs and injected with pristane	Humanized mice recapitulated key clinical and immunological features of SLE including production of human anti-nuclear autoantibodies, lupus nephritis, pulmonary serositis, decreased human lymphocytes in peripheral blood, hyperactivated B and T cells and increased proinflammatory cytokines	Gunawan et al. (2017)
SjS	NSG mice engrafted with PBMCs from patients with SjS	Mice engrafted with PBMCs from SjS patients had elevated levels of cytokines, particularly IFN-γ and IL-10. Histological analysis showed signs of inflammation within the lacrimal and salivary glands of mice engrafted with SjS. These infiltrates were mostly CD4^+^ and a small population of CD8^+^ T cells and B cells	Young et al. (2015)
Type I diabetes	NSG-Ab^o^ DR4 engrafted with CD4^+^ T cells pulsed with autoantigen-derived peptides	Mice injected with autoantigen-reactive CD4^+^ T cells lines from diabetic donors demonstrated human T cells infiltration into mouse islets, insulitis, and increased levels of demethylated β-cell–derived DNA in the bloodstream and reduced levels of insulin staining	Viehmann Milam et al. (2014)

*SLE* Systemic lupus erythematosus, *SjS* Sjogren’s syndrome, *CNS* central nervous system

### Graft-versus-host Disease

The occurrence of GvHD is a life-threatening complication that may develop following transplantations (Hu et al. 2011; Hu and Yang 2012). Even though GvHD has been intensively analyzed in non-humanized animal models, many human-specific mechanisms and treatments cannot be tested due to incongruence between humans and mice. Humanized mice are excellent substitutes to investigate exact human immune responses of GvHD and its related therapeutics (Ali et al. 2012; King et al. 2008; Kirkiles-Smith et al. 2009; Tobin et al. 2013; Wang et al. 2011; Zhao et al. 2015). An example of a humanized mouse model applied in GvHD studies is the engraftment of human PBMCs into immunodeficient mice (Ali et al. 2012). Post-transplantation, these mice demonstrated human lymphocytes infiltration into peripheral blood, spleen, lymph nodes, and BM of the mice, had enhanced tissue homing cells with a T-effector memory (T_EM_) phenotype and high levels of cutaneous lymphocyte antigen, recapitulating the exact pathogenesis of GvHD as observed in patients (Ali et al. 2012; Wang et al. 2011). Utilizing humanized mice to understand human-specific mechanisms of rejection provides a strong pre-clinical platform for the design of novel immunotherapies (Fogal et al. 2011; Onoe et al. 2011; Tobin et al. 2013), especially those targeting T_EM_ cell driven GvHD (Ali et al. 2012). Transplant rejection studies that have been conducted on humanized mice are listed in Table 7.

**Table 7:** GvHD modelled in humanized mice

GvHD	Models	Main findings	References
Cardiac tissue and skin	NSG mice were engrafted with human skin and artery tissue and injected with enriched human CD34^+^ HSC isolated from peripheral blood of G-colony stimulated factor pre-treated adults or PBMCs autologous to CD34^+^ donors either separately or together	Without T cells, CD14^+^CD68^+^ macrophages infiltrate allogeneic human skin but caused minimal injury and thrombosis. However, with the adoptive transfer of T cells autologous to HSC, CD14^+^CD68^+^ macrophages infiltrated allogeneic arterial interposition grafts, induced intimal expansion and calcification	Kirkiles-Smith et al. (2009)
hiPSCs	NSG mice engrafted with human fetal thymus and liver tissue under the kidney capsule and intravenously injected with autologous CD34^+^ human FL cells to create huBLT mice	Signs suggesting immune rejection of hiPSCs including formation of teratoma, infiltration of antigen-specific T cells and tissue necrosis were observed in these mice engrafted with autologous integration-free hiPSCs. In this study, autologous hiPSC-derived smooth muscle cells were highly immunogenic, while autologous hiPSC-derived retinal pigment epithelial cells were immune tolerated	Zhao et al. (2015)
Islet	NSG injected with human PBMCs and engrafted with human islets	Mice demonstrated low intra- and inter-donor variability of PBMCs engraftment. When treated with streptozotocin, mice were hyperglycemic but returned to normoglycemia when transplanted with islet cells. Upon injection of HLA-mismatched human PBMCs, mice showed signs of hyperglycemia, loss of human C-peptide, and rejection of human islet grafts	King et al. (2008)
PBMCs	NSG mice injected with human PBMCs alone or incubated with MSCs or stromal cells	Effectiveness of MSC therapy was dependent on the time of administration. Mice demonstrated signs of reduced liver and gut pathology and increased survival. MSC therapy did not result in donor T cell anergy and regulatory T cells did not induce the apoptosis of PBMCs; instead, it was associated with direct inhibition of donor CD4^+^ T cell proliferation and reduction of human TNF-α within the serum	Tobin et al. (2013)

*GvHD* graft-versus-host disease, *hiPSCs* human induced pluripotent stem cells, *PBMCs* peripheral blood mononuclear cells, *MSCs* mesenchymal stem cells, *HSC* hematopoietic stem cell, *TNF* tumor necrosis factor

## Human-Specific Drug Tests on Humanized Mouse Models

Non-human animal models are commonly used to test an array of human-specific therapeutics during pre-clinical trials. Due to a lack of human specificity, it is common for pre-clinical trials to inadequately identify exact pharmacokinetics, pharmacodynamics, and side-effects of therapeutics, which may result in debilitating and life-threatening situations when tested on humans (Horvath et al. 2012; Rehman et al. 2011; Xu et al. 2014). To improve from unsuccessful clinical trials, it is important to use validated and cost-effective animal models with high human specificity such as humanized mouse models to expand the traditional armamentarium of therapeutics for treatment of patients with complicated and progressive conditions.

Therapeutics successfully tested in mice with a functional human immune system includes an antiviral drug, peginterferon alpha-2a (Peg-IFNα2a) which demonstrated signs of HCV inhibition such as decreased human IFN-γ production, level of serum alanine aminotransferase, copies of HCV ribonucleic acid (RNA), and absence of leukocyte infiltration or fibrosis in the liver (Keng et al. 2015). Similar to clinical scenarios, humanized mice administered with Ipilimumab developed autoimmune disease with signs of weight loss, anti-nuclear antibodies, and adrenalitis. In addition, a biologic highly specific for human CD28, theralizumab, was tested in humanized mice engrafted with PBMCs (Weißmüller et al. 2016). These mice demonstrated severe reduction in CD45^+^ human cells, rapid drop of body temperature, elevated levels of cytokines, and succumbed to treatment within 6 h after antibody administration, recapitulating adverse effects observed in clinical scenarios (Weißmüller et al. 2016).

Considering the strengths, limitations, and potential developments of humanized mice, the current data indicate that these models are beneficial tools for researchers to investigate short and long-term studies of in vivo therapeutic interactions and toxicities to mitigate risks and ensure the safety of healthy volunteers and patients exposed to candidate agents during clinical trials. Therapeutics that has been tested on humanized mice is listed in Table 8.

**Table 8:** Therapeutics tested on humanized mice

Therapeutic	Alternative names	Model	Main findings	References
Alemtuzumab	Campath^®^, Campath-1H, MabCampath and Lemtrada	NSG mice intravenously injected with human PBMCs	Similar to clinical scenarios, Alemtuzumab induced severe temperature reduction in mice and bound to CD3 and CD52 but did not induce activation of markers CD25 and CD69	Brady et al. (2014)
ATG	Thymoglobulin^®^	NSG mice injected with human PBMCs	Mice that were given 150 µg of ATG intravenously became sick and were sacrificed within 1 h after treatment. Optimal dose of ATG in this study was 30 µg, where mice demonstrated mild clinical signs of drug treatment but recovered within 5 h	Brady et al. (2014)
Eltrombopag	Promacta^®^, Revolade	NOD/*scid* mice intravenously injected with human CD34^+^ UCB cells	Eltrombopag enhanced expansion and promoted multilineage hematopoiesis of HSPCs	Sun et al. (2012)
Ipilimumab	Yervoy^®^	Newborn NSG were intrahepatically injected with human CD34^+^ FL/UCB cells within 24 h of birth	Ipilimumab accelerated rejection of skin graft on humanized mice	Waldron-Lynch et al. (2012)
KM2760	–	NOG mice were engrafted with human PBMCs and injected with Hodgkin lymphoma cell line (L-428) or cutaneous T-cell lymphoma cell line (HH)	Anti-CCR4 mAb could be used to induce anti-tumor activity by removing CCR4-expressing tumors and downregulating regulatory T cells	Ito et al. (2009)
Lamivudine	3TC	C.B-17-*scid* engrafted with human thymus and liver tissues under the kidney capsule (*scid*-hu Thy/Liv mouse)	Relative to untreated mice, intraperitoneal injection of 3TC at 30 mg/kg/day had large reductions in viral RNA from a mean of 10^4.7^ to 10^1.8^ copies per 10^6^ cells	Stoddart et al. (2014)
Miltefosine	Impavido	Newborn NSG were engrafted with human CD34^+^ UBC cells and injected with stationary phase promastigote L. major into the footpad	Parasitic load was reduced and humanized mice demonstrated side-effects similar to clinical scenarios	Wege et al. (2011)
Muromonab-CD3	Orthoclone OKT3	NSG mice intravenously injected with human PBMCs	Administration of Muromonab-CD3, particularly intravenously resulted in cytokine storm and acute clinical symptoms such as piloerection, hypomotility and hypothermia	Brady et al. (2014)
Nivolumab	Opdivo^®^	RAG2^−/−^γc^−/−^ mice intravenously injected with human PBMCs	In mice engrafted with human colorectal HT-29 carcinoma cells and allogeneic human PBMCs, co-administration of Nivolumab and Urelumab slowed tumor growth	Sanmamed et al. (2015)
Oseltamivir	Tamiflu^®^	RAG2^−/−^γc^−/−^ mice intraperitoneally injected with H7N9	No therapeutic effects were observed when humanized mice were infected H7N9 were treated with Oseltamivir	Zheng et al. (2015)
Pamidronate	Aredia^®^	RAG2^−/−^γc^−/−^ mice intraperitoneally injected with H7N9	Pamidronate induced controlled viral replication and suppressed H7N9 injected within humanized mice. Treating mice with Pamidronate 3 days after infection could still ameliorate the disease	Zheng et al. (2015)
Peg-IFNα2a	Pegasys^®^	Newborn NSG were intrahepatically injected with human CD34^+^ FL cells within 72 h of birth	HCV copy numbers and serum ALT levels were reduced and no leukocyte infiltrations or fibrosis were observed in HCV-infected humanized mice intramuscularly injected with Peg*-*IFNα2a	Keng et al. (2015)
PG9	–	C.B-17-*scid* engrafted with human thymus and liver tissues under the kidney capsule (*scid*-hu Thy/Liv mouse)	PG9 provides minimal protective functions in *scid*-hu Thy/Liv mice challenged with HIV_NL4−3_. Antibodies can penetrate tissues to prevent infection	Stoddart et al. (2014)
PG16	–	NSG-BLT mice intravenously injected with human CD34^+^ FL cells	Single dose of PG16 administered a day before inoculation of HIV was effective in preventing infection	Stoddart et al. (2014)
Regorafenib	Stivarga^®^	Newborn NSG engrafted with patient primary AML cells	Regorafenib reduced the amount of engrafted human cells within the peripheral blood, extent of myeloid sarcoma and spleen size in mice injected with AML cells	Her et al. (2017)
Sorafenib	Nexavar^®^	Newborn NSG engrafted with patient primary AML cells	Sorafenib drastically reduced human cells in the peripheral blood, therefore, minimalising the extent of myeloid sarcoma and reducing spleen size in AML mouse model	Her et al. (2017)
Teplizumab	MGA031, hOKT3γ1(Ala-Ala)	Newborn NSG were intrahepatically injected with human CD34^+^ FL/UCB cells within 24 h of birth	Teplizumab delayed rejection of skin graft on humanized mice	Waldron-Lynch et al. (2012)
Theralizumab	TGN1412, CD28-SuperMAB and TAB08	NRG mice intravenously injected with human PBMCs	Similar to clinical scenarios, humanized mice had a rapid decrease in body temperature, became sick and succumbed to TGN1412, 2–6 h after antibody administration	Weißmüller et al. (2016)
Truvada (Combination of Tenofovir disoproxil fumarate and Emtricitabine)	–	C.B-17-*scid* engrafted with human thymus and liver tissues under the kidney capsule (*scid*-hu Thy/Liv mouse)	A large dose of Emtricitabine is results in only a small reduction of HIV RNA in HIV_JR−CSF_-challenged mice	Stoddart et al. (2014)
Urelumab	–	RAG2^−/−^γc^−/−^ mice intravenously injected with human PBMCs	Administration of both Urelumab and Nivolumab slowed tumor growth in mice engrafted with HT-29 colorectal carcinoma cells and allogenic human PBMCs	Sanmamed et al. (2015)

*ATG* anti-thymocyte globulin, *HSPCs* hematopoetic stem and progenitor cells

## Future Directions and Conclusion

To address gaps in humanized mice, scientists working in different biomedical disciplines are attempting a myriad of approaches including boosting human cell reconstitution, reducing graft rejections, supporting critical immune cell subsets, and improving human-specific responses toward pathogens to maximise the potential of humanized mice as a pre-clinical platform. Despite an optimistic outlook of humanized mice, there are considerable obstacles associated with the model that has to be solved as soon as possible. This includes scarce sources of human cells and tissues, particularly obtained from fetal samples due to ethical restrictions. A solution for this limitation is underway as teams around the world perfect induced pluripotent stem cell (iPSC) technology, which enables the use of patient-specific iPSCs allowing a renewable source of autologous cells sans immune rejection (Shi et al. 2017).

In humanized mice, secondary lymphoid structures are either missing or disorganised; this curtails essential humoral responses, resulting in impairments for both class switching and affinity maturation post-immunisation. To overcome this, lymphoid tissue inducer cells should be introduced without affecting IL2rg receptors (Lim et al. 2017). Alternatively, immunodeficient mice can be engrafted with both FL and cells that support FL cell growth from the same clinical donor and supplemented with cytokines (e.g., IL-1β, IL-2, IL-7, and GM-CSF), so that differentiation and maturation of HSCs can take place to improve functional immune cells including macrophages, follicular DC, and T helper cell reconstitution (Chen et al. 2009; Lim et al. 2017; Yong et al. 2016).

An absence of essential human cytokines hinders optimal HSC engraftment, differentiation, and maturation of functional immune cells. To tackle this issue, mouse models can be hydrodynamically boosted with plasmids encoding cytokines (Chen et al. 2009). Despite this improvement, binding of human cytokines may be hindered by residual mouse cytokines or may induce mouse cells to proliferate and displace the engraftment of human cells due to the cross-reactivity between some human and mouse cytokines. Eliminating this problem entirely would require absolute depletion of murine cells or the introduction of high affinity human-specific cytokines and growth factors.

Human cell engraftment is being negatively affected by mouse cells (RBCs and innate immune cells) that were not completely depleted during the construction of immunodeficient mice. To improve this, additional gene knock-outs could be added to current strains of immunodeficient mice to further reduce mouse RBCs, granulocytes and macrophage functions (Hu et al. 2011; Hu and Yang 2012), however, because of the low human erythrocyte engraftment, excessive reduction of mouse RBCs might result in anemic mice which has short lifespans, are weak and not suitable for experiments (Rongvaux et al. 2014). A long-term solution would be to optimise and increase the engraftment rate of human RBCs in humanized mice, so that all traces of mouse RBCs can be removed (Hu and Yang 2012).

Long-termism, critical analysis, and adequate troubleshooting to solve existing problems in humanized mice would undoubtedly provide exciting opportunities for the establishment of new and improved humanized models with increased human immune cell engraftment and enhanced functionality that would greatly benefit the community.
